# Using stochastic dynamic modelling to estimate the sensitivity of current and alternative surveillance program of *Salmonella* in conventional broiler production

**DOI:** 10.1038/s41598-020-76514-3

**Published:** 2020-11-10

**Authors:** Ofosuhene O. Apenteng, Mark E. Arnold, Håkan Vigre

**Affiliations:** 1 Division for Global Surveillance, Research Group for Genomic Epidemiology, National Food Institute, Technical University of Denmark, Kongens Lyngby, Denmark; 2 Animal and Plant Health Agency, College Road, Sutton Bonington, Loughborough, LE12 5RB UK

**Keywords:** Diseases, Health care

## Abstract

Since 2018, the EU commission has declared the Danish broiler industry to be *Salmonella* free. However, there is continuous *Salmonella* pressure from the environment, and a number of parent flocks and broiler flocks become infected annually. When a parent flock becomes infected, the infection can be transmitted vertically to the broiler flocks, before the parent flock is detected and destroyed, including the eggs at the hatchery. To address this issue, we developed stochastic dynamic modelling of transmission of *Salmonella* in parent flocks and combined that with the relation between flock prevalence and test sensitivity for environmental samples in the flock. Results suggested that after 10 and 100 infected hens were seeded, the likelihood of detecting an infected parent flock within the three first weeks after the infection was strongly influenced by the taking of five boot swabs (95% CI 70–100) instead of two (95% CI 40–100) or the supplementing of the two boot swabs by a dust sample (95% CI 43–100). Results suggest that the likelihood of detecting the broiler flock as infected in the program was estimated to at least 99% in broiler flock even if only one chicken was initially infected. These findings are of relevance for managing parent flocks and eggs at the hatchery in case of *Salmonella* infection in parent flocks in the Danish poultry.

## Introduction

In Denmark, the incidence in human salmonellosis increased rapidly in the second half of the 1980s because of the spread of *Salmonella* in broiler chickens^1^. Initially, a voluntary *Salmonella* control program was initiated^2^, and in 1996, the Ministry of Agriculture and Fisheries decided to implement a surveillance programme for the prevention of *Salmonella* in the broiler sector. In alignment with the EU legislation, the surveillance of *Salmonella* in the Danish broiler production system is based on the principle of top-down control, with a continuous monitoring program of *Salmonella* in parent flocks and hatcheries and testing of all broiler flocks before slaughter. At all stages, environmental samples are collected (boot swabs and dust) and sent to a laboratory, where the presence of *Salmonella* is determined by culture on selective media^3–5^. The current programme is presented in Table 1.

The conventional broiler production system is organised according to a production pyramid with a strict flow of animals from top to bottom, with grand-grand-...-grandparent flocks at the top and the broiler flocks in the bottom of the pyramid. The top of the pyramid consists of very few multinational companies supplying chickens to the parent flocks for the broiler production industry. In Denmark, day-old chickens of the parent flock are placed in rearing houses where they are reared to hens until an age of about 20 weeks of age. A few weeks before the hens start to produce hatching eggs, they are moved to egg-producing houses. The period of laying eggs for hatching is about 50 weeks, whereafter the parent flock is replaced. In Denmark, there are about 40 parent flocks, each with about 6000 hens producing approx. 6000 eggs per day^6,7^.

After collection and storage, the eggs are sent to one of the three large hatcheries in Denmark. After 3 weeks the eggs are hatched and the day-old chickens are transported to the broiler flocks. The broiler production takes place at about 200 farms producing about 120 million broilers for slaughter annually. The day-old chickens are delivered from one of the three large hatcheries in Denmark. Most broiler flocks have the size of 20,000–40,000 chickens, and usually, the chickens in a broiler house all originate from the same parent flock. It takes approximately one week for a parent flock to produce eggs/chickens to populate a broiler house. The broilers are slaughtered when they are 36–39 days old.

As a part of the biosecurity, a strict all-in/all-out procedure is practiced at all stages of the production with a total emptying, cleaning and disinfection before the facilities are populated with new flocks^8^.

**Table 1:** *Salmonella* surveillance programme for the adult flocks of the parent generation of the broiler production.

Adult flock	Samples taken	Current	Alternative	Taken by
Every 2nd week	Per flock	Hatcher basket liners from 5 baskets ($>1\, \mathrm{m}^{2}$ in total) or 10 g of broken egg-shells from each of 25 hatcher baskets (reduced to 25 g sub-sample). Analysed as one pool	removed	Owner-every 16th week DVFA
After each hatch	Per unit	Wet dust samples. Up to four hatchers of the same flock can be pooled	Wet dust samples. Up to four hatchers of the same flock can be pooled	Owner
Every week	Per unit	2 pairs of boot swabs (analysed as one pooled sample) or 1 faeces sample of 60 g	5 pairs of boot swabs (analysed as two pooled samples), or 2 pairs of boot swabs (analysed as one pooled sample) and 1 dust-sample, or 2 faeces samples consisting of 2 $\times$ 150 g	
0–4 weeks after moving, 8–0 weeks before slaughter	Per unit	5 pairs of boot swabs (analysed as two pooled samples), or 2 faeces samples consisting of 2 $\times$ 150 g	overlap with samples every week	DVFA

Alternative given by the Danish Veterinary Food Administration (DVFA)^8^.

In recent years, the reported infected flocks in the Danish broiler production has been very low, the sector being declared free of *Salmonella* by the EU commission since 2018. However, there is continuous *Salmonella* pressure from the environment, and every year, a number of parent flocks and broiler flocks become infected with *Salmonella*^8^. When a parent flock becomes infected, there is a risk of vertical transmission to broiler flock in the production pyramid. The experiences form outbreak investigation in the industry is that vertically infected broiler flocks are detected in the sampling taking place in the broiler flock between 16 and 26 days after insertion of day-old chickens using boot swabs, before the parent flock is detected as positive using boot swabs once a week. Typing results of *Salmonella* isolates from detected flocks has confirmed that vertical transmission from infected parent flocks to broiler flocks often takes place.

If zoonotic strains of *Salmonella* (e.g. *S*. Typhimurium, *S*. Enteritidis, monophasic *Salmonella*) are detected in a parent or broiler flock, the industry and the authorities have agreed that the flock should be killed and destroyed. In case of an infected parent flock, also eggs produced in the period between the last negative sample and the positive sample are destroyed at the hatchery. In relation to destroying eggs from a positive parent flock, there is large uncertainty about the length of time that the multiplication flock has been infected before it was detected. Therefore, there is no strong support for deciding whether only eggs produced in the period from the last negative sample (eggs produced during one week) or if all eggs at the hatchery from the flock (approx. eggs produced during 3 weeks) should be sent for destruction. This decision must balance between minimizing the likelihood of vertical transmission and economic compensation to the breeder. The cost for the industry for stamping out a *Salmonella* positive broiler flock is approximately 100,000€.

To obtain stronger support for this decision, the poultry industry and Danish food authorities asked us to estimate:

i. The likelihood that an infected parent flock is detected in the control program within the period of three weeks after infection, which is equivalent to the period the eggs are at the hatcheries before hatched and chickens are sent to broiler houses.
ii. The likelihood that a broiler flock that is infected due to vertical transmission is detected in the *Salmonella* monitoring program in broiler production.
iii. The effect of enhancing the collection of environment samples in the parent flock on the time between infection and detection of *Salmonella*.

In both cases, the estimates were obtained by modelling the transmission of *Salmonella* in the flock using dynamic compartment models. Subsequently, we estimated the sensitivity to detect *Salmonella* in a flock using repeated sampling of boot swabs taking into account the increased occurrence of *Salmonella* in the environment due to the spread of infection in the flock. The uncertainty in both transmission rates and the sensitivity of the diagnostic procedure was taken into account using stochastic modelling.

## Results

**Figure 1:**
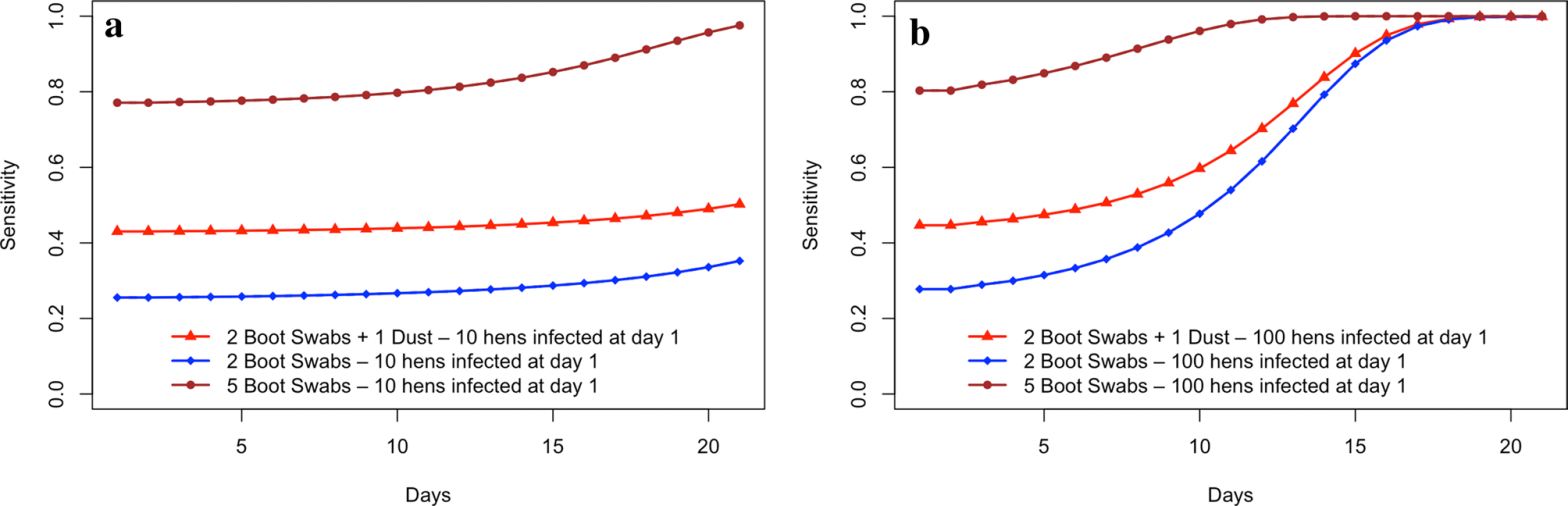
Estimated sensitivity to detect *Salmonella* using boot swabs with 10 and 100 hens infected at day 1. (**a**) Estimated sensitivity to detect *Salmonella* using boot swabs days 1 to 21 after infection in a multiplication flock of size 6000 hens (10 hens infected at day 1). The plotted lines show the estimated sensitivities using the most likely values of the parameters for transmission and sensitivity. (**b**) Estimated sensitivity to detect Salmonella using boot swabs days 1 to 21 after infection in a multiplication flock of size 6,000 hens (100 hens infected at day 1). The lines show the estimated sensitivities using the most likely values of the parameters for transmission and sensitivity.

Given the structure of the model, an increase in the prevalence of infected hens over time since introduction will result in a corresponding increase in the likelihood of classifying an infected flock over time since infection (see Supplementary Material). Therefore, the increase in prevalence over time since introduction will also result in a corresponding increase in the likelihood of classifying an infected flock as positive (detection sensitivity). The relationship between days since initial infection and the increase in detection sensitivity is illustrated in Fig. 1.

**Figure 2:**
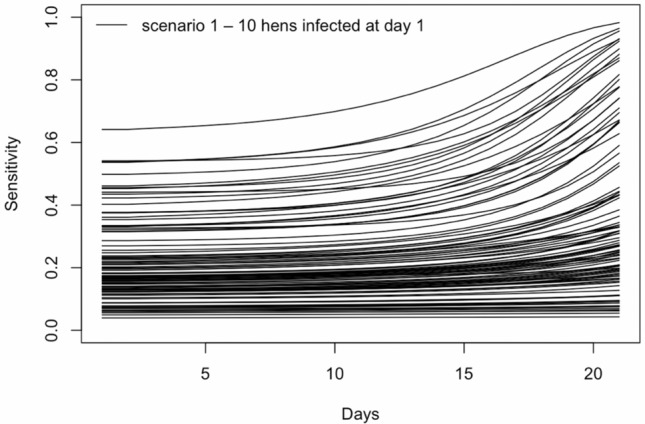
Estimated sensitivity to detect *Salmonella* using boot swabs days 1 to 21 after infection in a multiplication flock of size 6000 hens—each line represents the obtained results from an iteration using random values of the uncertainty parameters for transmission and sensitivity.

As a result of the uncertainties of the parameters for transmission and sensitivity, the uncertainty in the estimated sensitivity at different days was relatively large (see Fig. 2).

The 95% confidence interval of the likelihood of classifying a flock as positive at the first sampling after introduction, which occurs between day 1 and day 7 after *Salmonella* has been introduced into a parent flock, is given in Table 2.

**Table 2:** Estimated likelihood of classifying a multiplication flock of hens as positive at the first sampling (1 to 7 days after introduction) after *Salmonella* has been introduced into the flock.

Initial number of infected hens	Sampling method	6000 (1 week)		6000 (3 weeks)	
		Median (%)	95% CI	Median (%)	95% CI
10	2 Boot swabs	18	4–54	51	13–97
	2 Boot swabs + 1 dust	27	5–91	68	17–100
	5 Boot swabs	64	20–98	97	50–100
100	2 Boot swabs	23	6–63	95	40–100
	2 Boot swabs + 1 dust	32	6–94	98	43–100
	5 Boot swabs	73	24–99	100	70–100

**Figure 3:**
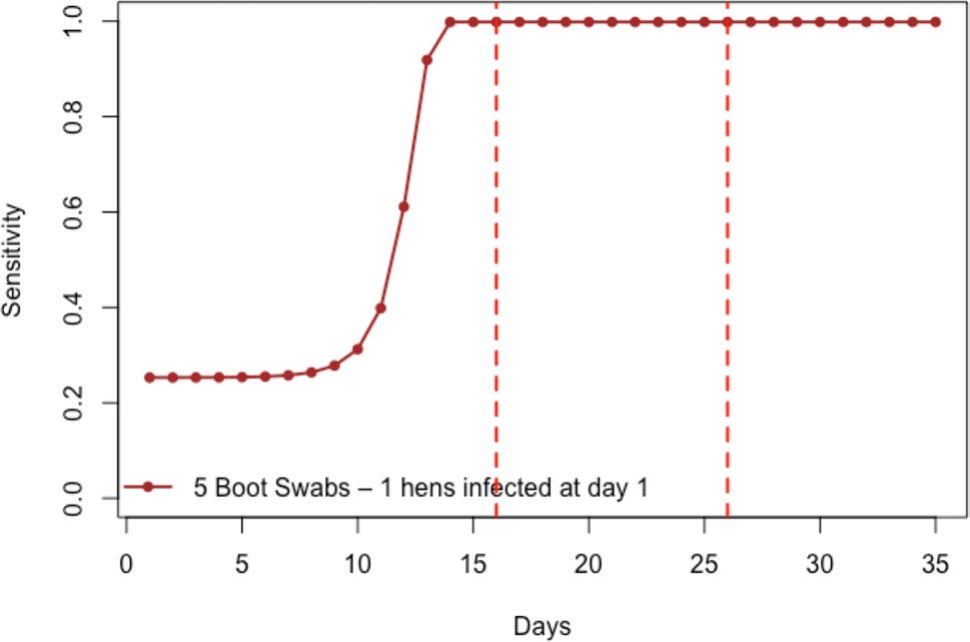
Estimated sensitivity to detect *Salmonella* using boot swabs days 1 to 35 after infection in a multiplication flock of size 40,000 hens. The plotted lines show the estimated sensitivities using the most likely values of the parameters for transmission and environmental sample sensitivity.

The likelihood of detecting a vertically infected broiler flock in the monitoring of broiler flock assuming one infected chicken when the broiler house is populated with day-old chickens was estimated to 100% (95%CI 99–100) as shown in Fig. 3.

## Discussion

Although the Danish broiler production has been classified as free from *Salmonella*, the parent and broiler flocks are continuously at risk of becoming infected by *Salmonella* from the surrounding environment. In the case of an infected parent flock, there is a risk of vertical transmission of *Salmonella* in the production to the broiler flocks. Both the Danish poultry industry and the Danish Veterinary and Food Administration share a common aim to reduce the risk of vertical transmission, and do this by adjusting the control program to detect infected parent flocks before the infection is transferred by eggs and chickens to the broiler flocks. A reliable model for predicting the effect of changing the sampling schedule in the *Salmonella* control program in the broiler production would, therefore, be highly valuable for guiding decision making.

The mathematical model used in this paper to described the spread of *Salmonella* within in a poultry flock a is modified version of the *SIR* model^9–11^ where the population only have two stages—susceptible and infected (for details see Supplementary Material). A similar approach of using an *SIR* model has recently been published by Collinaeu et al.^12^ who was modelling the transmission of *Salmonella* in a typical Canadian commercial broiler chicken flock.

The model for surveillance of *Salmonella* in the parent flocks in broiler production starts with a given number of initially infected hens, mimicking different routes of entry. There are several possible routes of entry, such as contaminated bedding material, feed, contact with wild animals, or introduction by human activity. In all cases that have been observed in parent flocks in the Danish production the last years, outbreak investigations indicate that the infection has been present for several weeks in the parent flock before detection, which indicates that only very few hens were initially infected in the parent flock. However, the actual route of entry has not been determined, and therefore there is no definite advice for how to reduce the frequency of introduction of *Salmonella* into the production system.

The subsequent transmission in the flock is modelled using a *SI* dynamic model, with a decline in the transmission rate over time for each infected hen. In the initial phase of the modelling, we used a fixed transmission rate for an infected hen. Using a non-declining transmission rate resulted in very fast spread within the flock, reaching almost 100% within few days, which is unrealistic when compared to empirical data which indicates that *Salmonella* can be present in a parent flock for several weeks before reaching a relatively high prevalence. The uncertainties in the parameters used for modelling this decline was taking into account using stochastic modelling, and combined with the uncertainties in the estimation of the sensitivity, there is relatively large uncertainty in the likelihood to detect the infection at different point of times after infection. However, for a relative assessment of the effect of changing the sampling schedule, the model can be used to predict the relative effect of changing sampling schedule, whereas an absolute prediction is very uncertain.

It has been shown that the rate of transmission between hens and broilers vary between *Salmonella* strains^13–15^. The parameters for the transmission rates that we used in the model were obtained from transmission studies of *S*. Enteritidis. Although *S*. Enteritidis historically has been one of the most relevant strains of *Salmonella*, other zoonotic strains have become increasingly important in the poultry industry such as *S*. Typhimurium and monophasic variant of *S*. Typhimurium, but also other strains. It cannot be assumed that all strains have the same transmission rate. Strains that are not specifically adapted to broilers might have a slower spread, or even not be able to establish an infection in a flock. Subsequently, the likelihood to detect these strains after introduction into a flock will probably be lower relative to the likelihood to detect *S*. Enteritidis. On the other hand, the lower infection prevalence in these strains will result in less onward spreading of *Salmonella*.

In reality, the spread of infections within a flock varies very much between farms due to multiple management factors. The variation in transmission rates between farms has not been integrated into stochastic modelling, so the estimated sensitivities are based on the assumption that the transmission of *Salmonella* in farms is relatively similar. Thus, the estimated sensitivity of surveillance should be viewed as the mean sensitivity of the surveillance program and not as the absolute sensitivity of each case when *Salmonella* is introduced into a parent flock. Therefore, the estimated sensitivity should be used for decision support and not for estimating the likelihood of detecting an infection in a particular farm.

The estimated confidence intervals of detecting the infection at the initial phase of the spread were relatively wide. The size of the confidence intervals originates from the uncertainty in the transmission rate and the function describing how sensitivity depends on the flock prevalence. When the prevalence increases due to spread, the uncertainty in the likelihood to detect the infection originating from the transmission rate and sensitivity function decrease. In the current surveillance system there is no false positive results, because in case of positive samples confirmative sampling and testing are performed. In accordance to this, in the model the specificity in the surveillance system was fixed to 100%.

Estimation of the sensitivity of environmental sampling to detect an infected flock according to the infection prevalence was calculated utilizing results and equations obtained in a study performed in the British non-cage egg-layer production^16–18^. Although several factors might cause differences in the dynamics of a *Salmonella* infection in a parent flock compared to egg-layer production, and there could be possible differences in the performance of the environmental sampling between the different sectors^19^, the generalization of the prevalence-dependent equation to estimate the sensitivity of boot swabs and dust sample, respectively, is independent of a possible difference in the infection dynamic between the production systems.

The results from our model suggests that with the current sampling approach, *Salmonella* can be present and spread in a multiplication flock several weeks before the flock tests positive from an environmental sample. In this period, eggs sent to hatcheries can be infected or contaminated with *Salmonella*, and *Salmonella* can be transferred to the broiler flock by day old chicken. The likelihood of detecting an infected multiplication flock within three weeks after infection depends very much on the number of initially infected hens, which depends on the route of introduction.

In case of vertical transmission to a broiler flock, the likelihood of detecting the broiler flock as infected in the program was estimated to be almost 100%. This is despite assuming only one infected day-old chicken in the whole flock of 40,000 chickens. This indicates that even if there is a likelihood of vertical transmission from multiplication flock to broiler flock, the likelihood that the broiler flock is detected before being sent to slaughter (and consumption) is at least 99% taking know uncertainties of the modelling parameters into account.

The result obtained from the model when assessing the current sampling schedule is very much in alignment with observed data from the current surveillance program. During 2017–2018, one parent flock was infected with *S*.Enteritidis and one parent flock with *S*.Typhimurium. In both cases, the occurrence of *Salmonella* in the breeding pyramid was first detected in the broiler flocks.

We also used the model to assess the effect of alternative sampling schedules, which all included more frequent sampling and more samples. These alternatives all have a higher sensitivity at any given prevalence. The approach where sampling for surveillance in a population is simulated simultaneously with the simulation of spread of the infection of interest in the population can be generalized for assessing surveillance programs of infectious diseases in any population. By simultaneously simulating the spread of the disease in the population and random (or directed sampling) sampling within the population it is possible to estimate the performance of a program under realistic conditions. By stochastic simulation, the uncertainty in knowledge to spread of infection and the sensitivity of diagnostic procedure was integrated in the results. Knowledge of the overall uncertainty of surveillance programs is important for comparing the performance between different sampling schedules.

## Methods

In this section, we describe the estimation of the sensitivity of the control program in the multiplication flock. The estimation of the sensitivity in the broiler flock is briefly described in the end of the section.

### Modelling the spread of *Salmonella* in a flock of hens using compartment model

**Figure 4:**
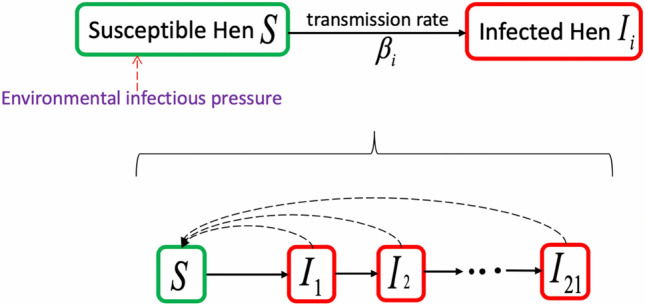
Schematic representation of the $SI_i$ compartment model within a parent flock, $i=1,2,\ldots ,21$. Solid arrows represent transitions between compartments; dotted arrows represent dependencies of transmission rates between each infected compartments and susceptible compartment.

The spread of *Salmonella* in a flock was modelled using a dynamic model, each individual in a flock is either in a susceptible (*S*) compartment or an infected (*I*) compartment. The unit of time in the *SI* model was one day, and the outcome of the *SI* model was the proportion of infected birds each day after initial infection, should *Salmonella* be introduced from the environment into a parent flock (Fig. 4). In the model, we assumed that if an animal became infected it was infected for the rest of the time (no recovery). Previous studies of transmission of *Salmonella* within flocks has shown that the infectiousness of infected hens decline over time after infection^20–23^. This was integrated into the model by using multiple compartments of infected cases ( $I_i$), where *i* representing the number of days after infection ($i=1,2,\ldots ,21$), and the infectiousness ($\beta _i$) represent the decline in infectiousness ($\beta _1,\beta _2,\ldots ,\beta _{21}$).The infectiousness was estimated using the equation (1) in Thomas et al.^24^ (for details see Supplementary Material). We assumed that the model had homogeneous mixing between all individuals in the flock. That is, susceptible hens acquire infection following contact with an infectious hen with a force of infection $\lambda =S/N\sum \beta _i I_i$, where $\beta _i$ is the product of the contact rate and the probability of strain infection. *N* is the total number of hens in the population, and finally, $\delta$ is the movement from one infected compartment to other infected compartments. At the end each day, the number of infected hens (*I*) was divided by the number of hens in the population to obtain the day-specific prevalence. A pictorial representation of the model is shown in see Fig. 4. It was assumed that the latent period from infection to that a hen is starting to become infectiousness was 1 day^24^.

The flow of hens from one compartment to another is frequency-dependent (mass-action) and is modelled using ordinary differential equations (for details see Supplementary Material). The model parameters for the dynamical model are taken from the previous work of Thomas et al.^24^ based on *Salmonella* Enteritidis as shown in Table 3. The dynamic model was coded in mc2d package in R using the described model in Fig. 4 (for the detailed description of the model see equation (1–3) in the Supplementary Material).

**Table 3:** Parameter estimates and transmission analysis from day 1 to day 21 of a mathematical model representing transmission within a Danish broiler flock.

Parameter	Description	Value$^{\mathrm{a}}$	Source
$\delta$	Movement between the infected compartment	1 day$^{-1}$	Assumed
$\gamma$	Rate of decline of environmental infectiousness	0.170	^24^
$\beta _1$	Transmission rate between *S* and $I_1$	0.470	^24^
$\beta _2$	Transmission rate between *S* and $I_2$	0.397	Estimated
$\beta _3$	Transmission rate between *S* and $I_3$	0.336	Estimated
.	.	.	.
.	.	.	.
.	.	.	.
$\beta _{21}$	Transmission rate between *S* and $I_{21}$	0.022	Estimated

^a^The presented values are the mean of the estimated parameters.

### Current and alternative sampling and testing methods

**Figure 5:**
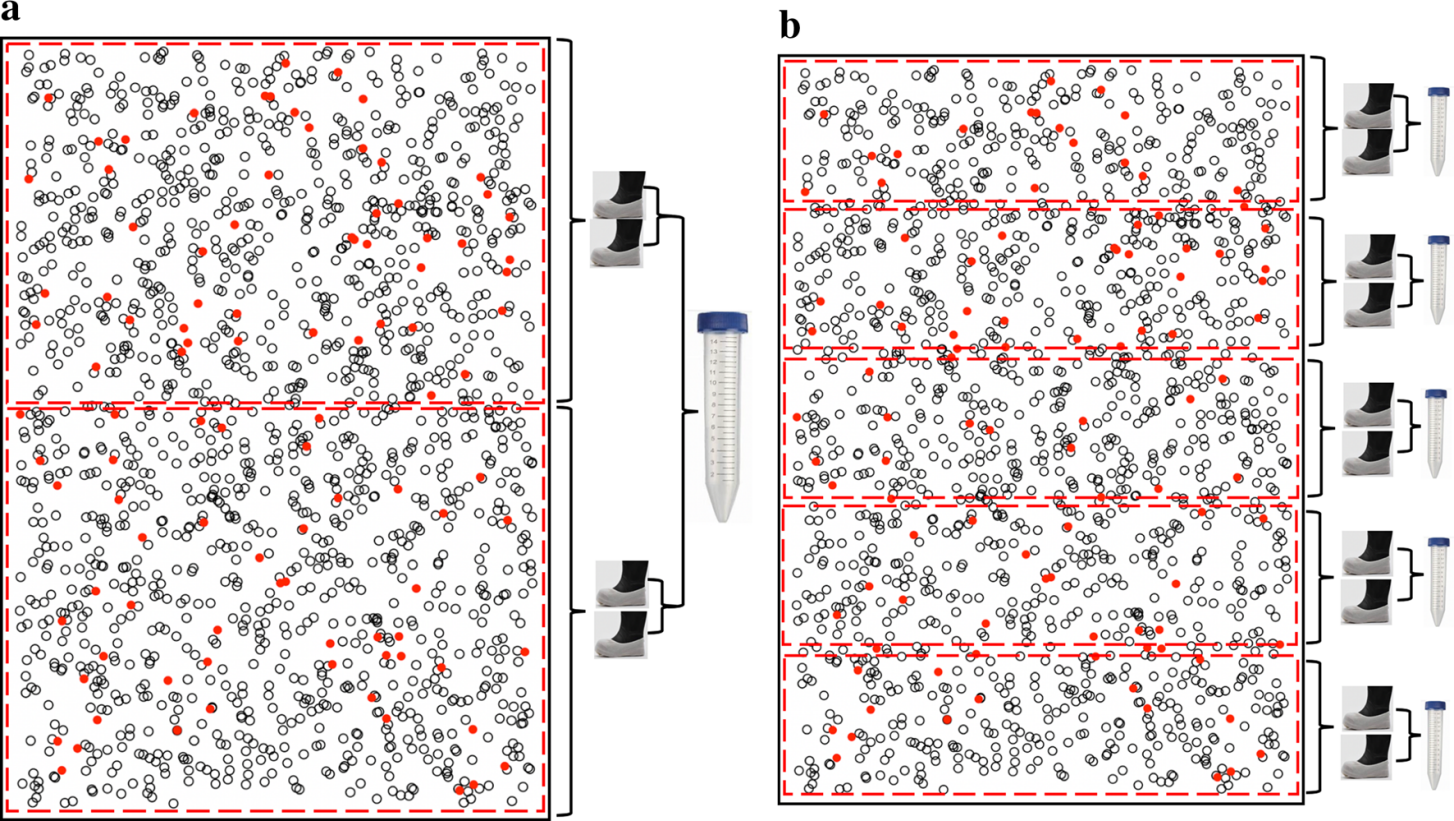
Current testing methods. (**a**) Two pairs boot swabs (analysed as one pooled sample) - sampling in parent flocks. Red dots represent droppings from infected animals and black dots non-infected animals. (**b**) Five pairs boot swabs—sampling in broiler flocks. Red dots represent droppings from infected animals and black dots non-infected animals.

In the current *Salmonella* control program, the sampling of environmental samples from the parent flocks takes place every week. The procedure for sampling in the parent flock is that the house is divided into two parts and two pairs of boot swabs are collected by walking randomly in each part of the house. The two pairs of boot swabs are pooled into one tube which is sent to the laboratory for further analysis (Fig. 5a). In the broiler flock, the sampling of environmental samples from the parent flocks takes about 16 and 10 days before slaughter. The procedure in the broiler flock is that the house is divided into five equal parts. Five pairs of boot swabs are collected by walking randomly in each of the five sections. Each pair of both swabs is sent to the lab for further analysis (Fig. 5b) (see^16^ for further details).

The Danish Food and Food Veterinary Administration has suggested an alternative method for the weekly sampling in the parent flock which is equivalent to the sampling of 5 pairs of boot swabs that is used in the broiler flock given in Table 1, as shown in Fig. 5.

### Scenarios used in the modelling and simulation for layers

The modelling and simulation for *Salmonella* in the parent flock were performed in different scenarios of flock size (total number of hens in the population, $$N = 6000$$ and 12, 000) and number of infected hens the first day of infection (10 and 100 hens) to investigate how the detection sensitivity is influenced by variation in these factors. The flock size of parent flocks in Denmark range between approximately 6000 and 12,000 hens. We modelled two scenarios for the number of infected hens the first day the day of infection: 10 and 100, respectively, representing low and high initial levels of seeding of the population to mimic different seed values of *Salmonella* into the parent flock. In the transmission model, the prevalence of infected hens any day after introduction was estimated as the sum of hens in compartment$$ I_1$$ to $$I_{21}$$ divided by *N*.

### Estimating the prevalence dependent sensitivity of the monitoring systems

The monitoring of *Salmonella* in poultry production is based on environmental sampling of faeces using boot swabs and bacteriological culture in selective media. The possibility that a boot swab results in a positive laboratory result are dependent on the house prevalence of hens infected by *Salmonella*. Studies performed in the UK^16,18^, has shown that this dependency of sensitivity on prevalence can be described using the function:

1 $$\begin{aligned} \text{ sensitivity }=\frac{\exp (\alpha +\rho \times \text{ prevalence})}{\exp (1+\alpha +\rho \times \text{ prevalence})}, \end{aligned}$$

where $\alpha$ is the intercept and $\rho$ represents the dependence of the method on the house prevalence.

In the multiplication flock, boot swabs samples are collected every week. The likelihood of classifying a flock as infected according to the time of sampling after the entry of *Salmonella* into the flock was estimated as the mean of the sensitivity of detection at day $1, 2, \ldots ,7$ after introduction. The likelihood of obtaining a positive laboratory result from an infected flock increases over time since the introduction of *Salmonella*, due to spread of infection in the flock and hence an increased amount of *Salmonella* in the environment. Therefore, the likelihood of classifying a flock as positive within the three first weeks after introduction of *Salmonella* was estimated using the formula:

2 $$\begin{aligned}&\text{ overall } \text{ sensitivity}_t=1-(1-\text{ sensitivity}_t) \times (1-\text{ sensitivity}_{t+7})\nonumber \\&\quad \times (1-\text{ sensitivity}_{t+14}), \end{aligned}$$

where *t* is the number of days between the introduction of *Salmonella* and the first day of sampling (1–7).

**Figure 6:**
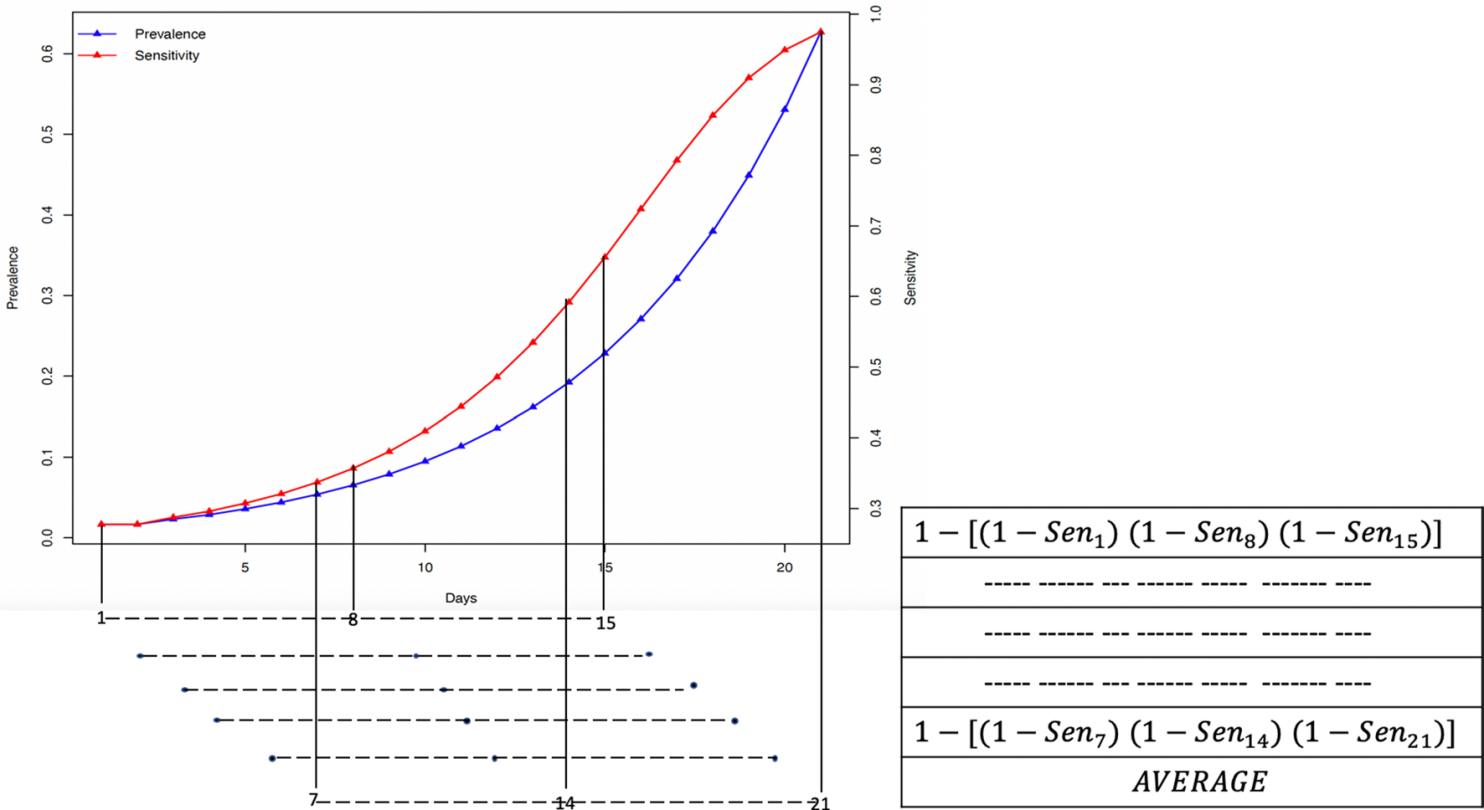
Sampling method for detecting a positive flock within first three weeks after introduction of *Salmonella*into the flock.

The daily probability of infection being introduced into the flock is equal for days 1 to 21, and therefore the likelihood of classifying an infected flock positive with the first three sampling points after the introduction ($\text{ sensitivity}_{3weeks}$) is estimated as the $\overline{\text{ overall } \text{ sensitivity}_t}$ for $t = 1{-}21$. For more information as how this sampling calculation was done, see Fig. 6.

### Estimating the uncertainty in $\text{ sensitivity}_{3weeks}$ due to uncertainty in transmission rates and sensitivity

The uncertainty in the parameters used in the transmission model and the estimation of sensitivity was taking into account in the estimations of the sensitivity by Monte Carlo simulation using 1001 iterations. Based on^24^, the uncertainty of the transmission rate, $\beta$ and rate of decline of environmental infectiousness, $\gamma$ was expressed as *N*(0.47, 0.105) and *N*(0.17, 0.039) respectively. Based on^16,18^ and Mark Arnold (personal communication) the uncertainty of the parameters $\rho$ and $\alpha$ in (1) were expressed as *N*(10.65, 3.765) and $N(-1.53, 0.81)$ respectively. Using Monte Carlo, the $\text{ sensitivity}_{3weeks}$ was estimated using 1001 iterations—each iteration with varying values of the uncertain parameters. The 95% confidence interval of the $\text{ sensitivity}_{3weeks}$ was obtained using the 2.5% and 97.5% percentile of the distribution of the 1001 estimates of $\text{ sensitivity}_{3weeks}$.

### Scenarios used in the modelling and simulation for broilers

The modelling and simulation for *Salmonella* in the broiler flock with the total number of hens in population of 40,000 broiler chicken and one infected case. A time step of one day was used and the disease progression was simulated for 21 and 35 days in case of the parent flocks and broiler flocks, respectively. The estimation of the sensitivity in broiler flock was following the same procedure as for hens, assuming initially one infected chick at day one, a transmission rate of 1.15 (95% CI 0.76–1.75)^25^, and collecting of boot swabs at day 16 and 26 after insertion into the house. The coding of the Monte Carlo simulation was done in mc2d package in R.

## Supplementary information

Supplementary Information.

Supplementary material 2

Supplementary material 3

Supplementary material 4

## References

[1] Wegener HC (2003). Salmonella control programs in Denmark. Emerg. Infect. Dis..

[2] Bisgaard M (1992). A voluntary Salmonella control programme for the broiler industry, implemented by the Danish Poultry Council. Int. J. Food Microbiol..

[3] Bailey J (2001). Sources and movement of Salmonella through integrated poultry operations: a multistate epidemiological investigation. J. Food Prot..

[4] Carrique-Mas J (2008). Sampling and bacteriological detection of Salmonella in poultry and poultry premises: a review. Rev. Sci. Tech..

[5] Soria MC (2017). Salmonella spp. contamination in commercial layer hen farms using different types of samples and detection methods. Poult. Sci..

[6] Rosenquist H, Nielsen NL, Sommer HM, Nørrung B, Christensen BB (2003). Quantitative risk assessment of human campylobacteriosis associated with thermophilic campylobacter species in chickens. Int. J. Food Microbiol..

[7] Pedersen M, Thamsborg S, Fisker C, Ranvig H, Christensen J (2003). New production systems: evaluation of organic broiler production in Denmark. J. Appl. Poult. Res..

[8] Helwigh, B., Petersen, C. K. & Müller, L. Annual report on zoonoses in Denmark 2018. DTU Food (2018).

[9] Kermack WO, McKendrick AG (1927). A contribution to the mathematical theory of epidemics. Proc. R. Soc. Lond. Ser. A.

[10] Kermack WO, McKendrick AG (1932). Contributions to the mathematical theory of epidemics. ii.—the problem of endemicity. Proc. R. Soc. Lond. Ser. A.

[11] Kermack WO, McKendrick AG (1933). Contributions to the mathematical theory of epidemics. iii.—further studies of the problem of endemicity. Proc. R. Soc. Lond. Ser. A.

[12] Collineau L (2020). A within-flock model of Salmonella Heidelberg transmission in broiler chickens. Prev. Vet. Med..

[13] Liljebjelke KA (2005). Vertical and horizontal transmission of Salmonella within integrated broiler production system. Foodbourne Pathog. Dis..

[14] Thiagarajan D, Saeed A, Asem E (1994). Mechanism of transovarian transmission of *Salmonella enteritidis* in laying hens. Poult. Sci..

[15] Cason J, Cox N, Bailey J (1994). Transmission of *Salmonella typhimurium* during hatching of broiler chicks. Avian Dis..

[16] Arnold M, Martelli F, McLaren I, Davies R (2014). Estimation of the sensitivity of environmental sampling for detection of Salmonella in commercial layer flocks post-introduction of national control programmes. Epidemiol. Infect..

[17] Arnold M, Carrique-Mas J, Davies R (2010). Sensitivity of environmental sampling methods for detecting *Salmonella enteritidis* in commercial laying flocks relative to the within-flock prevalence. Epidemiol. Infect..

[18] Arnold M, Martelli F, McLaren I, Davies R (2014). Estimation of the rate of egg contamination fromSalmonella infected chickens. Zoonoses Public Health.

[19] Skov MN, Carstensen B, Tornøe N, Madsen M (1999). Evaluation of sampling methods for the detection of Salmonella in broiler flocks. J. Appl. Microbiol..

[20] Braden CR (2006). *Salmonella enterica* serotype enteritidis and eggs: a national epidemic in the United States. Clin. Infect. Dis..

[21] Holt PS, Geden CJ, Moore RW, Gast RK (2007). Isolation of salmonella enterica serovar enteritidis from houseflies (*Musca domestica*) found in rooms containing *Salmonella serovar* enteritidis-challenged hens. Appl. Environ. Microbiol..

[22] Gast RK, Holt PS (1998). Persistence of *Salmonella enteritidis* from one day of age until maturity in experimentally infected layer chickens. Poult. Sci..

[23] Foley SL (2011). Population dynamics of *Salmonella enterica* serotypes in commercial egg and poultry production. Appl. Environ. Microbiol..

[24] Thomas M (2009). Quantification of horizontal transmission of *Salmonella enterica *serovar enteritidis bacteria in pair-housed groups of laying hens. Appl. Environ. Microbiol..

[25] Heres L, Urlings H, Wagenaar J (2004). Transmission of Salmonella between broiler chickens fed with fermented liquid feed. Epidemiol. Infect..

