# Supplementary material for: Using stochastic dynamic modelling to estimate the sensitivity of current and alternative surveillance program of Salmonella in conventional broiler production
Source: Sci Rep. 2020 Nov 10;10:19441. doi: 10.1038/s41598-020-76514-3 (PMC7655952; doi:10.1038/s41598-020-76514-3)
Supplement: Supplementary file 1 — Supplementary Information. [file 41598_2020_76514_MOESM1_ESM.pdf]

Using stochastic dynamic modelling to estimate the sensitivity of current and alternative surveillance program of *Salmonella* in conventional broiler production

Ofosuhene O. Apenteng[1,*,+], Mark E. Arnold[2], and Håkan Vigre[1,+]

[1]Division for Global Surveillance, Research Group for Genomic Epidemiology, National Food Institute, Technical University of Denmark, Kongens Lyngby, Denmark. ofap@food.dtu.dk & hvig@food.dtu.dk
[2]Animal and Plant Health Agency, College Road, Sutton Bonington, Loughborough, LE12 5RB, UK. Mark.Arnold@apha.gov.uk
[*]corresponding.author:ofap@food.dtu.dk
[+]these authors contributed equally to this work

ABSTRACT

This document provides supplementary information on "Using probabilistic uncertainty analysis of dynamic modelling to estimate the sensitivity of the monitoring program of Salmonella in multiplication flocks in the Danish broiler industry". Here we provide the detailed of the mathematical expression based on our model and other figures illustrating various results in the main paper.

The flow of hens from one compartment to another of frequency-dependent (mass-action) are described by the ordinary differential equations for details see supplementary.

$$\frac{dS}{dt} = -\frac{S}{N}\sum_{i}^{n}\beta_i I_i, \tag{1}$$

$$\frac{dI_1}{dt} = \frac{S}{N}\sum_{i}^{n}\beta_i I_i - \delta n I_1, \tag{2}$$

$$\frac{dI_i}{dt} = \delta n I_{i-1} - \delta n I_1 \qquad \forall_i = 2,...,n, \tag{3}$$

where n is the number of stages in the infected period, where the movement between compartments is multiply by δ to maintain the average infected period even when the number of compartment changes.

As described the main paper. The infectiousness was estimated using the equation (1) in Thomas et al, is given by:

$$\beta_i(\tau) = \begin{cases} 0 & \text{if} \quad \tau < 1 \\ \beta_i & \text{if} \quad 1 \le \tau < \tau_1 \\ \beta_i \exp[-\gamma(\tau - \tau_i)] & \text{if} \quad \tau \ge \tau_1 \end{cases}$$

Where β_i represents all the mean values and denoted by β, which is defined as the mean initial colonization rate per day. Similarly, the mean of all colonization rates is presented as $[\beta_i(\tau)]$ abd denoted by $\beta_i(\tau)$.
Figure give the representation of the prevalence curve use to predict the other values in the main article for seeded cases of 10 hens and 100 hens infected at the day 1, respectively.

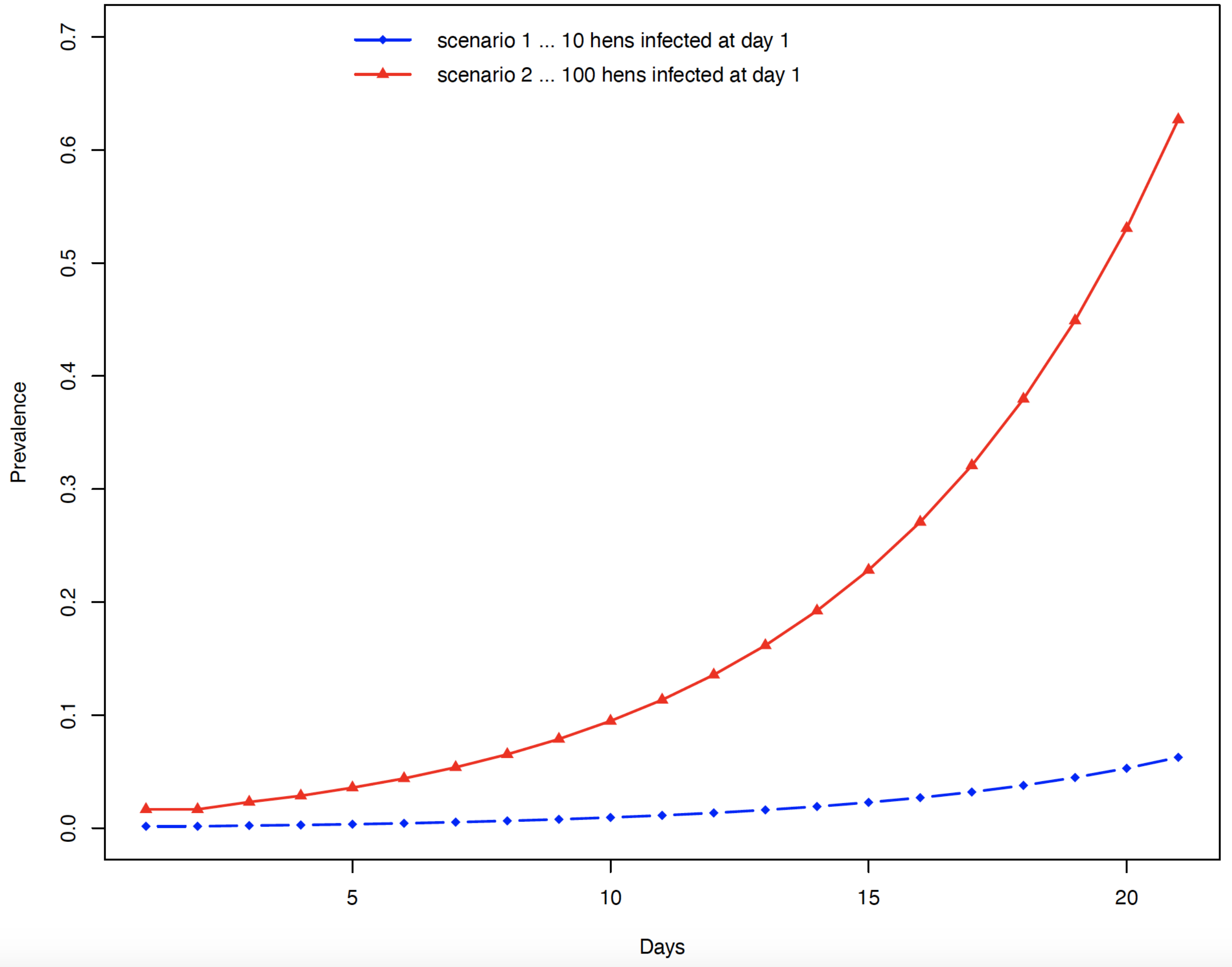

Figure 1. Estimated prevalence of Salmonella using boot swabs days 1 to 21 after infection in a multiplication flock of size 6,000 hens. The plotted lines show the estimated prevalence using the most likely values of the parameters for transmissionk.

Therefore, the increase in prevalence over time since introduction will also result in a corresponding increase in the likelihood of classifying an infected flock as positive (detection sensitivity), for sample size of 12,000 hens as well. The relationship between days since initial infection and the increase in detection sensitivity is illustrated in Figure 1.

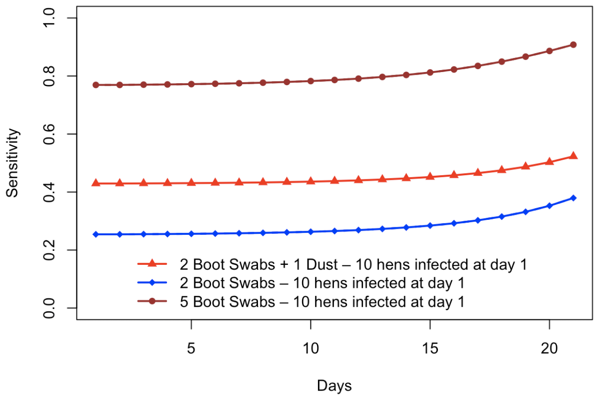

(a) Estimated sensitivity to detect *Salmonella* using boot swabs days 1 to 21 after infection in a multiplication flock of size 12,000 hens (10 hens infected at day 1). The plotted lines show the estimated sensitivities using the most likely values of the parameters for transmission and sensitivity.

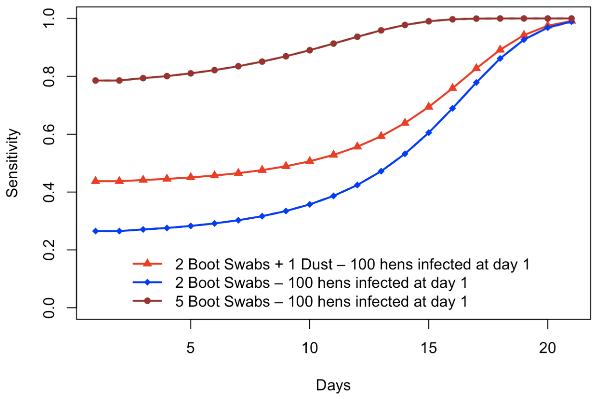

(b) Estimated sensitivity to detect *Salmonella* using boot swabs days 1 to 21 after infection in a multiplication flock of size 12,000 hens (100 hens infected at day 1). The lines show the estimated sensitivities using the most likely values of the parameters for transmission and sensitivity.

Figure 2. Estimated sensitivity to detect *Salmonella* using boot swabs with 10 and 100 hens infected at day 1.

The 95% confidence interval of the likelihood of classifying a flock as positive at the first sampling after introduction, which is at day 1 to 7 after *Salmonella* has been introduced into a parent flock, is given in Table 1.

		12000 (one week)		12000 (three weeks)	
Initial number of infected hens	Sampling Method	Median	95% CI	Median	95% CI
10	2 Boot Swabs	18%	4-54%	50%	14-92%
	2 Boot Swabs + 1 Dust	27%	5-91%	64%	16-100%
	5 Boot Swabs	63%	20-98%	96%	49-100%
100	2 Boot Swabs	20%	5-59%	80%	25-100%
	2 Boot Swabs + 1 Dust	29%	5-92%	89%	27-100%
	5 Boot Swabs	68%	22-99%	100%	59-100%

Table 1. Estimated likelihood of classifying a multiplication flock of hens as positive within the three first events of sampling after Salmonella has been introduced into the flock.

Acknowledgements

This work was supported by funding from the European Union's Horizon 2020 Research and Innovation programme under grant agreement No 773830: One Health European Joint Programme.

Author contributions statement

O.O.A. and H.V. conceived and designed the framework, O.O.A. wrote the code. O.O.A. and H.V. analysed the data, and did the calculations. O.O.A. and H.V. and M.E.A. wrote the paper. O.O.A. and H.V. edited the manuscript. All authors reviewed and edited the draft and gave final approval for publication.
