# Supplementary figures and images for: Using stochastic dynamic modelling to estimate the sensitivity of current and alternative surveillance program of Salmonella in conventional broiler production
Source: Sci Rep. 2020 Nov 10;10:19441. doi: 10.1038/s41598-020-76514-3 (PMC7655952; doi:10.1038/s41598-020-76514-3)

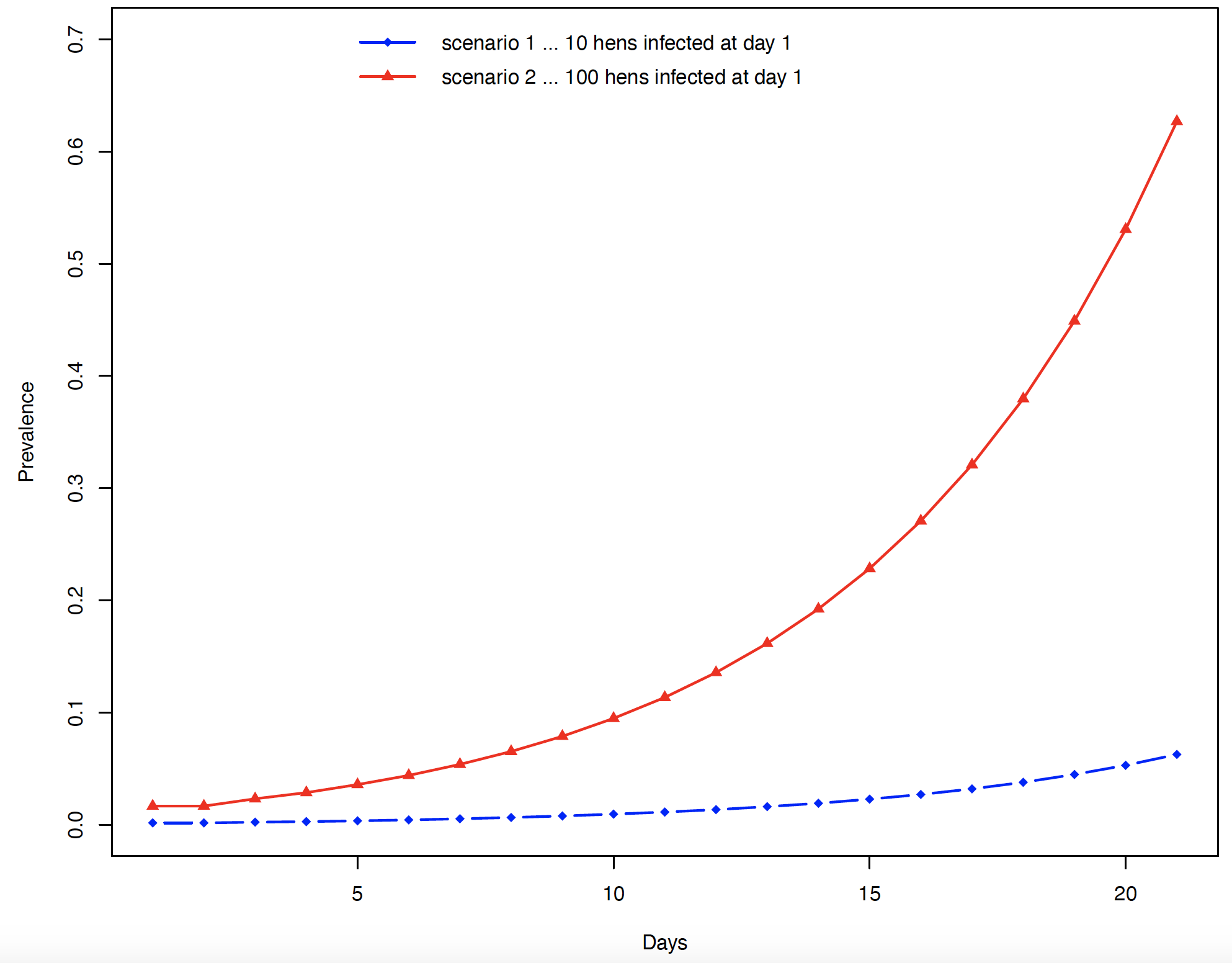

Supplement: Supplementary file 2 — Supplementary material 2 [file 41598_2020_76514_MOESM2_ESM.png]

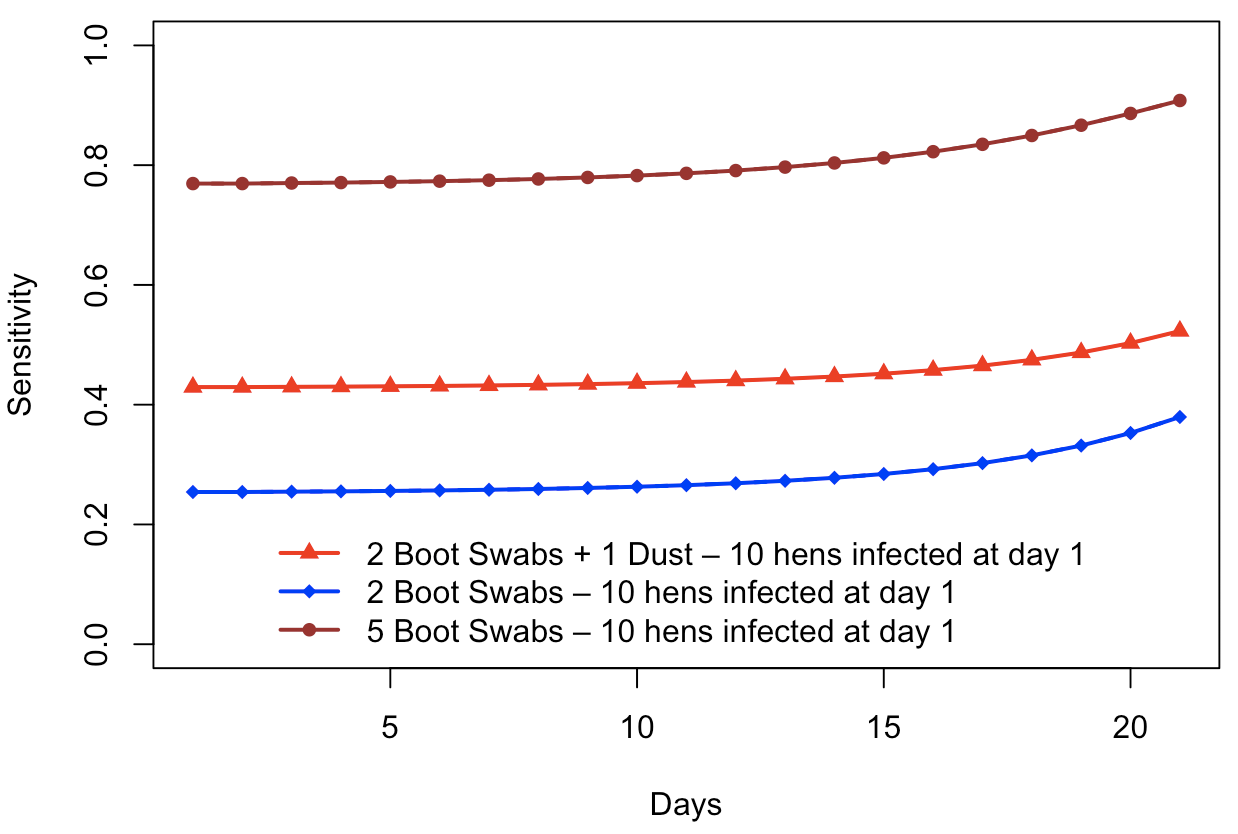

Supplement: Supplementary file 3 — Supplementary material 3 [file 41598_2020_76514_MOESM3_ESM.png]

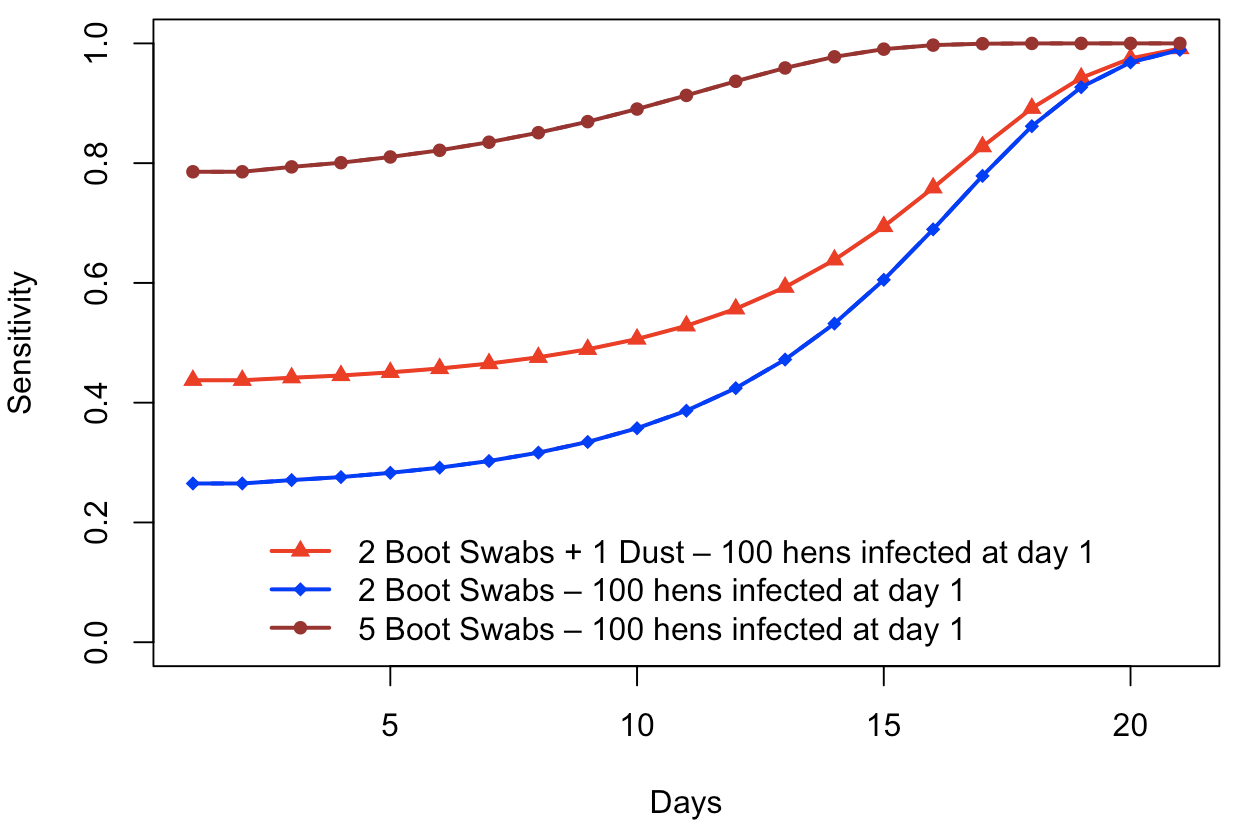

Supplement: Supplementary file 4 — Supplementary material 4 [file 41598_2020_76514_MOESM4_ESM.png]
